# Gene networks for three feed efficiency criteria reveal shared and specific biological processes

**DOI:** 10.1186/s12711-020-00585-z

**Published:** 2020-11-10

**Authors:** Sébastien Taussat, Mekki Boussaha, Yuliaxis Ramayo-Caldas, Pauline Martin, Eric Venot, Gonzalo Cantalapiedra-Hijar, Chris Hozé, Sébastien Fritz, Gilles Renand

**Affiliations:** 1 Université Paris-Saclay, INRAE, AgroParisTech, GABI, 78350 Jouy-en-Josas, France; 2 Allice, 75012 Paris, France; 3 INRAE, Université Clermont Auvergne, Vetagro Sup, UMRH, 63122 Saint-Genès-Champanelle, France

## Abstract

**Background:**

French beef producers suffer from the decrease in profitability of their farms mainly because of the continuous increase in feed costs. Selection for feed efficiency in beef cattle represents a relevant solution to face this problem. However, feed efficiency is a complex trait that can be assessed by three major criteria: residual feed intake (RFI), residual gain (RG) and feed efficiency ratio (FE), which involve different genetic determinisms. An analysis that combines phenotype and whole-genome sequence data provides a unique framework for genomic studies. The aim of our study was to identify the gene networks and the biological processes that are responsible for the genetic determinism that is shared between these three feed efficiency criteria.

**Results:**

A population of 1477 French Charolais young bulls was phenotyped for feed intake (FI), average daily gain (ADG) and final weight (FW) to estimate RFI, RG and FE. A subset of 789 young bulls was genotyped on the BovineSNP50 single nucleotide polymorphism (SNP) array and imputed at the sequence level using RUN6 of the 1000 Bull Genomes Project. We conducted a genome-wide association study (GWAS) to estimate the individual effect of 8.5 million SNPs and applied an association weight matrix (AWM) approach to analyse the results, one for each feed efficiency criterion. The results highlighted co-association networks including 626 genes for RFI, 426 for RG and 564 for FE. Enrichment assessment revealed the biological processes that show the strongest association with RFI, RG and FE, i.e. digestive tract (salivary, gastric and mucin secretion) and metabolic processes (cellular and cardiovascular). Energetic functions were more associated with RFI and FE and cardio-vascular and cellular processes with RG. Several hormones such as apelin, glucagon, insulin, aldosterone, the gonadotrophin releasing hormone and the thyroid hormone were also identified, and these should be tested in future studies as candidate biomarkers for feed efficiency.

**Conclusions:**

The combination of network and pathway analyses at the sequence level led to the identification of both common and specific mechanisms that are involved in RFI, RG and FE, and to a better understanding of the genetic determinism underlying these three criteria. The effects of the genes involved in each of the identified processes need to be tested in genomic evaluations to confirm the potential gain in reliability of using functional variants to select animals for feed efficiency.

## Background

Improving feed efficiency of beef cattle is a major concern for beef producers. In France, on the one hand, the carcass weight of Charolais beef bulls has increased by 13% between 1996 and 2016 [1, 2] due to the improvement of management and breeding practices and on the other hand, the consumption of concentrate has risen by 29% and feeding costs by 50% over the same period [3]. Depending on the economic weight of the inputs and outputs, feed efficiency can be improved by reducing consumption without affecting production and/or, conversely, by increasing production without increasing consumption. The simplest measure to estimate the feed efficiency of growing animals is the feed efficiency ratio (FE), which is the average daily gain (ADG) divided by feed intake (FI) or its inverse, the feed conversion rate (FCR = FI/ADG). However, FE is difficult to use as a selection criterion because the genetic responses of the components (FI and ADG) of this ratio are unpredictable [4]. Currently, the most commonly used criterion for selecting efficient animals is residual feed intake (RFI), which is the difference between observed FI and FI predicted from the animal’s maintenance and needs [5]. Usually, for growing animals, RFI is calculated as the residual of the regression of intake on metabolic body weight and daily gain. This criterion is not correlated with its components, and animals that have negative RFI values, i.e. that eat less than expected, are considered as efficient. Another feed efficiency criterion that has been suggested to identify differences in feed use among growing animals is residual gain (RG) [5], which is the difference between observed and predicted ADG and is calculated as the residual from a multiple regression of ADG on metabolic body weight and feed intake. Animals with higher RG values are more efficient.

Estimates of the heritability of FCR, RFI and RG are moderate, i.e. on average 0.23, 0.33 and 0.28, respectively (see review in [6]). Thus, genetic improvement of the feed efficiency of growing cattle can be achieved by breeding high merit animals. However, implementation of a breeding program based on performance records and pedigree information is difficult, because measuring individual feed intake is too costly and time-consuming for routine recording on commercial farms. In this context, genomic selection can be a relevant alternative to improve feed efficiency, since it requires phenotype records for the reference population only. Thus, it is important to identify genetic markers associated with feed efficiency. Several genome-wide association studies (GWAS) have been conducted on cattle populations and have revealed putative quantitative trait loci (QTL) that are associated with phenotypic differences in feed use and associated traits [7–15]. These studies confirmed that many genes are involved in the genetic differences regarding feed use, each one explaining only a small proportion of the phenotypic variance and most of them being breed-specific. By using single nucleotide polymorphisms (SNPs) that are significantly associated with performance, genomic estimated breeding values (GEBV) for feed efficiency can now be predicted, but with a moderate accuracy [16]. The use of whole-genome sequence (WGS) data increases the accuracy of GEBV of complex traits [17], thus allowing the detection of causative variants [18]. Since the inclusion of causative variants can improve the accuracy of genomic predictions and allows robust predictions across cattle populations, the identification of functional genes involved in feed efficiency will greatly benefit programs that aim at improving this trait [19]. The identification of causative variants in WGS is still very difficult since a large number of variants can be in linkage disequilibrium (LD) within a QTL region and imputation of WGS for rare variants is far from perfect [20]. The recent development of systems biology approaches allows the identification of the relationships between markers, genes and phenotypes, and can enhance our knowledge on the genetic architecture of complex traits [21–24].

A previous study [25] that estimated genetic parameters of French Charolais beef bulls showed that FE, RFI and RG had similar and moderate heritabilities (0.35 to 0.36) and were moderately to strongly correlated: i.e. genetic correlation estimates were − 0.77 between FE and RFI, − 0.45 between RFI and RG, and 0.91 between FE and RG. Such genetic correlations suggest that part of the genetic determinism underlying RFI, RG and FE is shared, but that different genetic mechanisms are also involved in the expression of each trait. The objective of our work was to identify some of the biological processes that are responsible for both the shared and specific genetic determinisms among these three feed efficiency criteria. First, we performed a GWAS of imputed WGS data for each criterion and the associated traits. Then, we applied an association weight matrix (AWM) approach to identify candidate gene networks and biological processes with an enrichment analysis.

## Methods

### Animal management and phenotyping

During this experiment, all animals were kept indoors, handled with care following the Institut National de Recherche pour l’Agriculture, l’Alimentation et l’Environnement (INRAE) ethics policy in accordance with the guidelines for animal research of the French Ministry of Agriculture (https://www.legifrance.gouv.fr/eli/decret/2013/2/1/2013-118/jo/texte).

The animal design used in this study was similar to that previously reported in [25]. Briefly, 60 Charolais bulls were used to inseminate purebred Charolais females on the INRAE experimental farm in Bourges. Progenies were born between 1988 and 2009 and weaned at 221 ± 3 days, on average. Immediately after weaning, uncastrated male calves were moved to a fattening barn and allotted by groups of seven individuals into pens, which were equipped with Calan Gates (American Calan, Northwood, NH) that allow individual recording of feed intake. These young bulls were adapted to the fattening diet during 6 to 8 weeks and were then fed ad libitum with a complete pelleted diet that is composed of 29% dehydrated alfalfa hay, 29% dehydrated beet pulp, 21% bran and other ingredients to make a balanced diet. At the beginning of the test period, young bulls were on average 275 (± 10) days old and were weighed on two consecutive days to establish their initial body weight. Then, they were weighed every 14 days to monitor their growth. All the young bulls were tested until 15 months of age and, for half of the animals, feeding was continued until 19 months of age. At the end of the test period, they were weighed on two consecutive days to establish the final weight (FW).

### Description of the traits measured

Records for growth and feed intake traits in the experimental fattening barn were available for 1477 animals. Initial and final weights were used to compute $\text{ADG}$ and mid-test weight. The metabolic mid-test weight ($\text{MMW}$) was calculated as the mid-test weight^0.75^. Daily $\text{FI}$ of the fattening bulls was equal to the mean of all daily dry matter intake records over the period. Feed intake was used to calculate $\text{RFI}$, $\text{RG}$ and the $\text{FE}$ ratio. Residual feed intake ($\text{RFI}$) was equal to the difference between observed and expected $\text{FI}$, computed by a regression of $\text{FI}$ on $\text{MMW}$ and$\text{ADG}$, using the Proc GLM of SAS/STAT® software, version 9.4 of the SAS System for Linux (Copyright© 2002 to 2012 by SAS Institute Inc., Cary, NC, USA). The model was:

$${\text{FI}} = year + \beta 1 \left( {{\text{MMW}}} \right) + \beta 2 \left( {{\text{ADG}}} \right) + \beta 3 \left( {\text{final age}} \right) + {\text{RFI,}}$$

where $year$ was the fixed effect of the contemporary group (from 1988 to 2009), $\beta 1$ was the partial regression of $\text{FI}$ on $\text{MMW}$, $\beta 2$ was the partial regression of $\text{FI}$ on $\text{ADG}$, $\beta 3$ was the partial regression of $\text{FI}$ on age at the end of the test. Residual gain ($\text{RG}$) was the difference between $\text{ADG}$ and expected $\text{ADG}$ and was computed by a regression of $\text{ADG}$ on $\text{MMW}$ and $\text{FI}$, using the Proc GLM of SAS/STAT® software. The model was:

$$\text{ADG} = year + \beta 1 (\text{MMW}) + \beta 2 (\text{FI}) + \beta 3 (\text{final age}) + \text{RG}$$

where $year$ was the fixed effect of the contemporary group (from 1988 to 2009), $\beta 1$ was the partial regression of $\text{ADG}$ on $\text{MMW}$, $\beta 2$ was the partial regression of $\text{ADG}$ on $\text{FI}$, $\beta 3$ was the partial regression of $\text{ADG}$ on age at the end of the test. $\text{FE}$ was equal to $\text{ADG}/\text{FI}$ and represented the gain in body weight for 1 kg of feed consumed.

### 50 K genotypes and imputation to whole-genome sequences

A subset of 789 young bulls was genotyped with the BovineSNP50™ BeadChip (50 K) (Illumina Inc., San Diego, CA). Means and standard deviations of feed efficiency and production traits for this subset are in Table 1. After quality control based on the French national evaluation system [26], i.e. removing an individual with a call rate higher than 95%, a SNP with a call rate higher than 90% or with a minor allele frequency (MAF) higher than 5% in at least one major French dairy cattle breed, and SNPs with a deviation of genotype frequencies from Hardy–Weinberg equilibrium with P > 10^–4^, 43,801 autosomal SNPs remained for further analyses.

**Table 1:** Means and standard deviations (SD) of traits studied in all young bulls (n = 1447) and in the sub-population of genotyped young bulls (n = 789)

Traits	Whole population^a^		Genotyped sub-population^b^	
	Mean	SD	Mean	SD
RFI	0.00	0.81	− 0.01	0.81
RG	0.00	0.14	0.00	0.13
FE	0.14	0.02	0.14	0.02
FI	10.62	1.31	10.63	1.23
FW	682	87	695	87
ADG	1.45	0.20	1.44	0.19

*RFI* residual feed intake, *RG* residual gain, *FE* feed efficiency ratio, *FI* daily feed intake, *FW* final weight, *ADG* average daily gain

^a^Results for the whole population from Taussat et al. [25]

^b^Subset of the terminal bulls that are genotyped and used for GWAS analysis

The genotypes of the young bulls were imputed to WGS as described in [27]. Briefly, this approach includes two steps: imputation from 50 to 777 K high-density (HD) SNPs and then to WGS. With this method, the accuracy of the WGS imputed variants is higher [28]. For the first step, we used the FImpute software [29] to perform imputations from 50 K to HD using a within-breed reference set of 664 Charolais bulls that were genotyped with the Illumina BovineHD BeadChip (Illumina Inc., San Diego, CA). For the second step, we used the Minimac software [30] to perform imputation from HD density to WGS, using WGS variants from the 6th run (UMD 3.1 assembly) of the 1000 Bull Genomes consortium. This run contained 2333 *Bos taurus* individuals from different cattle breeds, most of them purebred. However, to reduce the time necessary for imputation to WGS, a subset of 18 cattle pure breeds was selected to build the reference population, by ensuring a sufficient representativeness of each breed and the genetic proximity with the French cattle pure breeds [see Additional file 1]. The new reference population panel included 1466 WGS purebred individuals of which 128 were Charolais individuals.

The accuracy of the imputation from HD to WGS was assessed by the coefficient of determination (R^2^) as calculated by the Minimac software. To remove variants with the lowest accuracies of imputation, only those with a R^2^ higher than 30% and a MAF higher than 1% were retained for further association analyses, i.e. 8,602,123 variants.

### Association analysis

Single-trait association analyses were performed between the 8,602,123 variants and RFI, RG, FE, FI, FW and ADG of young bulls. These phenotypes were precorrected using the Proc GLM of SAS for the fixed effects of contemporary group (22 years), age of the dam (3, 4, 5, 6 years and more) and twinning (single or twin), and age at the end of the test was included as a covariate. All association analyses were computed with the GCTA software (version 1.26) [31], using the *mlma* option, and applied to a mixed linear model with the following formula:

$$\mathbf{y} = \mathbf{1}\upmu + \mathbf{x}\text{b}+ \mathbf{u} + \mathbf{e}$$

where $\mathbf{y}$ is the vector of corrected phenotypes; ${\upmu}$ is the overall mean; $\mathbf{1}$ is a vector of ones; $\text{b}$ is the z-score of the additive effect of the SNP analysed; $\mathbf{x}$ is the predicted dosage (ranging from 0 to 2); $\mathbf{u}\sim N(\mathbf{0},\mathbf{G}{\upsigma }_{\mathbf{u}}^{2})$ is the vector of random polygenic effect, where $\mathbf{G}$ is the genomic relationship matrix, calculated using the 50 K SNP genotypes, and ${\upsigma }_{\mathbf{u}}^{2}$ is the polygenic variance that is estimated based on the null model ($\mathbf{y}={\upmu}+\mathbf{u}+\mathbf{e}$), and then fixed when the association between each variant and the trait of interest is tested; and $\mathbf{e}\sim N(\mathbf{0},\mathbf{I}{\upsigma }_{\mathbf{e}}^{2})$ is the vector of random residual effects, where $\mathbf{I}$ the identity matrix and ${\upsigma }_{\mathbf{e}}^{2}$ the residual variance.

### Network analysis

We applied the AWM approach [21] to detect co-associated genes by combining the results of the GWAS with the network inference algorithms. Each AWM procedure was performed using RFI, RG or FE as key phenotype. First, a $\text{n}\times \text{m}$ matrix that contained z-score standardized additive effects with SNPs row-wise ($\text{n}$= 8,602,123) and phenotypes column-wise ($\text{m}$= 6) was built. In this first step, only the SNPs with a P-value ≤ 0.001 for the key phenotype were included in the matrix. Then, correlation coefficients were calculated between the z-scores of SNP effects for the key phenotypes and those of the five other traits. Traits that were correlated (|r|≥ 0.25) were kept for the next step, which aimed at detecting new SNPs associated (P-value ≤ 0.001) with at least two other traits among the remaining ones and including them in the AWM matrices. The last step of the AWM procedure consisted in selecting one marker per gene. First, SNPs that were located within the nearest annotated gene (UMD 3.1 assembly) were identified, but since in general we found several SNPs within each identified gene, the SNP that was associated with the largest number of traits or, in case of a tie, with the lowest cumulated P-value over the associated traits, was selected.

The proportion of variance explained by the SNPs retained after the three AWM analyses was estimated using the GCTA software. To do that, a new genomic relationship matrix was computed for each feed efficiency trait with SNPs from the AWM analysis only. Then, these SNPs were used to perform a genome-based restricted maximum likelihood (GREML) analysis to estimate the SNP-based heritability for each trait [32]. This analysis was repeated with a similar number of randomly selected WGS SNPs to compare the heritabilities estimated from AWM SNPs and from random SNPs.

### Enrichment analysis

Enrichment in gene ontology (GO) terms and pathways from the Kyoto Encyclopedia of Genes and Genomes (KEGG) was investigated for the genes selected by the AWM approach. The plug-in ClueGO 2.5.5 [33] for Cytoscape 3.7.1 was used for this analysis with a selection of levels 3 to 8 of the GO hierarchy to obtain neither too general nor too specific GO terms. A gene set was considered to be enriched if the P-value associated with the hypergeometric test was lower than 0.05, after application of the Benjamini–Hochberg correction for multiple testing. GO terms and KEGG pathways were subsequently clustered in functional groups if the kappa statistic was higher than 0.4. The results from ClueGO were used in CluePedia 1.5.5 [34], a plug-in for Cytoscape, to visualize the network interaction between genes and enriched processes.

## Results

For feed efficiency traits, the Manhattan plots from the GWAS are in Figure S1 [see Additional file 2: Fig. S1]. A Bonferroni threshold, which accounts for multiple testing, is commonly used as significance threshold for such GWAS analyses. For our data, this threshold would have been really high, i.e. reaching 8.24 (− log_10_(0.05/8,602,123)), because of the huge number of markers and tests at the sequence level. Considering this very stringent threshold, no variant exceeded this threshold for the three GWAS performed here. However, in a first approach, the 100 variants with the lowest P-value for RFI, RG and FE were selected to find the most likely associated genes (variants included in the genes). This analysis revealed seven genes for RFI, eight for RG and 11 for FE (see Table 2). Of these 26 genes, two were associated with both RFI and FE (*CAPN7* and *CACNA1E*) and two with both RG and FE (*ENSBTAG00000030623* and *MROH7*). No gene was associated with both RFI and RG.

**Table 2:** Genes associated with SNPs from the 100 SNPs with the lowest p-value for each feed efficiency trait

BTA	Gene symbol	Gene name	Trait affected^a^
1	*CAPN7*	*Calpain 7*	RFI, FE
1	*SH3BP5*	*SH3 domain binding protein 5*	RFI
3	*INSL5*	*Insulin like 5*	RG
3	*TCTEX1D1*	*Tctex1 domain containing 1*	RG
3	*ENSBTAG00000030623*		RG, FE
3	*MROH7*	*Maestro heat like repeat family member 7*	RG, FE
5	*GUCY2C*	*Guanylate cyclase 2C*	FE
5	*SAMM50*	*SAMM50 sorting and assembly machinery component*	FE
10	*SLC35F4*	*Solute carrier family 35 member F4*	FE
11	*SULT6B1*	*Sulfotransferase family 6B member 1*	RFI
11	*NCK2*	*NCK adaptor protein 2*	RG
15	*UBQLNL*	*Ubiquilin like*	RG
15	*MMP13*	*Matrix metallopeptidase 13*	RFI
16	*CACNA1E*	*Calcium voltage-gated channel subunit alpha1 E*	RFI, FE
17	*TRIM2*	*Tripartite motif containing 2*	RFI
17	*KNTC1*	*Kinetochore associated 1*	RG
18	*LOC514658*	*Bile salt sulfotransferase*	FE
18	*ENSBTAG00000040054*		FE
22	*GADL1*	*Glutamate decarboxylase like 1*	FE
24	*ZNF407*	*Zinc finger protein 407*	FE
27	*RBPMS*	*RNA binding protein*	RG
28	*CHAT*	*Choline O-acetyltransferase*	RFI

^a^Trait abbreviations: *RFI* residual feed intake, *RG* residual gain, *FE* feed efficiency ratio

### Association matrix

All the genes retained after the AWM analyses are in Tables S1, S2 and S3 [see Additional file 3: Tables S1, S2 and S3]. In the AWM analysis with RFI as key phenotype, all the traits were correlated (using SNP z-scores) with RFI (|r|≥ 0.25). Thus, the six traits were used in the AWM analysis, which decreased the number of variants from 8,602,123 to 15,977 SNPs among which 5761 were located in 626 genes (Table 3 and [see Additional file 3: Table S1]). Among these 626 genes, 231 were associated with RFI only and 155 were associated with RFI and at least one other trait (82 genes with FE, 53 with FI, 14 with both FE and FI, 6 with both RG and FE). The remaining 240 genes were associated with at least two traits other than RFI: 66 genes with RG and ADG, 57 genes with RG and FE and 48 genes with FI and FW. Overall, for the feed efficiency traits, this AWM analysis highlighted 386 genes for RFI, 179 for FE and 146 for RG and for the production traits, 133 genes for FI, 92 for FW and 130 for ADG (see Table 4).

**Table 3:** Number of genes retained for the three association weight matrix (AWM) analyses

Key phenotype (KP)	RFI	RG	FE
Number of traits retained	5	4 (not FI)	5
Number of genes specific to the KP	231	142	169
Number of genes common between the KP and traits	155	146	179
Number of other genes	240	138	216
Total number of genes retained	626	426	564

*RFI* residual feed intake, *RG* residual gain, *FE* feed efficiency ratio

**Table 4:** Number of genes associated with traits for the three association weight matrix (AWM) analyses

	Traits	Key phenotype		
		**RFI**	**RG**	**FE**
Number of genes associated with traits	RFI	386	101	155
	RG	146	288	146
	FE	179	174	348
	FI	133	NA	133
	FW	92	44	92
	ADG	130	127	130

*RFI* residual feed intake, *RG* residual gain, *FE* feed efficiency ratio, *FI* daily feed intake, *FW* final weight, *ADG* average daily gain

The AWM analysis with RG as key phenotype decreased the number of variants from 8,602,123 after the GWAS to 11,169 SNPs. Feed intake was not correlated (using SNP z-scores) with RG and was not in the AWM analysis. Of the 11,169 SNPs, 3750 that were located in 426 genes were retained (Table 3 and [see Additional file 3: Table S2]). Among these 426 genes, 142 were associated with RG only and 146 with RG and at least another trait: 67 with ADG, 56 with FE, 15 with both FE and ADG, two with both FW and ADG, one with FE, FW and ADG, and five with both RFI and FE. Among the 138 remaining genes, 96 were associated with RFI and FE, 41 with FW and ADG, and one with FE and ADG. This AWM analysis showed that 288 genes were associated with RG, 174 with FE, 127 with ADG, 101 with RFI, 44 with FW and none with FI (see Table 4).

In total, 13,751 SNPs were selected after the AWM analysis with FE as key phenotype. All the traits were correlated (using SNP z-scores) with FE and used in the AWM analysis. Among the 13,751 SNPs, 4139 were located in 564 genes (Table 3 and [see Additional file 3: Table S3]) among which 169 genes were identified with FE only and 179 with FE and at least one another trait: 82 with RFI, 57 with RG, five with FI, 14 with both RFI and FI, 14 with both RG and ADG, one with RG, FW and ADG and six with both RFI and RG. Among the 216 remaining genes, 66 were detected with both RG and ADG, 53 with both RFI and FI and 48 with both FI and FW. Overall, for feed efficiency traits, this AWM analysis highlighted 348 genes for FE, 155 with RFI and 146 with RG and for production traits, 133 genes for FI, 92 for FW and 130 genes for ADG (Table 4).

The RFI, RG and FE AWM analyses highlighted the same five genes that were associated with the three feed efficiency criteria: *WDR27*, *PCDH8*, *CDCP2*, *ENSBTAG00000030623* and *MROH7* [see Additional file 3: Tables S1, S2 and S3]. A sixth gene (*NCK2*) was identified with RFI, RG and FE in the AWM analyses with RFI or FE as key phenotype and with RG, FE and ADG in the AWM analysis with RG as key phenotype.

After each AWM analysis, genomic correlations were calculated using the variant’s z-score of the additive effects of the retained variants and compared with the genetic correlations computed from pedigree data (see Table 5). The genomic correlations between RFI, RG and FE were similar regardless of the AWM analyses. However, genomic correlations between RFI and FE were stronger (− 0.91 to − 0.93) than genetic correlation (− 0.78), which suggests that the selected variants explained an important proportion of the genetic relationships between RFI and FE. Although the estimated genomic correlations between RG and FE were lower than the genetic correlation, their high values showed that a large number of variants were shared between RG and FE. Genomic correlations between RFI and RG confirmed that the association between these two feed efficiency criteria was the weakest, which suggests that different mechanisms are involved in the expression of these two traits.

**Table 5:** Genomic correlations calculated using additive effects of the variants selected by the three association weight matrix (AWM) analyses (above the diagonal) and genetic correlations estimated from [25] (below the diagonal)

Traits	RFI	RG	FE	FI	FW	ADG
RFI		− 0.57^a^	− 0.93	0.88	− 0.10	− 0.11
		− 0.53^b^	− 0.91	NA	− 0.22	− 0.29
		− 0.60^c^	− 0.93	0.85	− 0.01	− 0.21
RG	− 0.45		0.78	− 0.26	0.47	0.85
			0.81	NA	0.67	0.82
			0.83	− 0.27	0.55	0.94
FE	− 0.78	0.91		− 0.75	0.43	0.62
				NA	0.24	0.37
				− 0.70	0.18	0.49
FI	0.77	− 0.16	− 0.49		0.37	0.30
					NA	NA
					0.52	0.26
FW	0.10	0.28	0.09	0.76		0.86
						0.84
						0.82
ADG	− 0.04	0.80	0.57	0.44	0.80	

*RFI* residual feed intake, *RG* residual gain, *FE* feed efficiency ratio, *FI* daily feed intake, *FW* final weight, *ADG* average daily gain

^a^AWM using RFI as key phenotype

^b^AWM using RG as key phenotype

^c^AWM using FE as key phenotype

### SNP-based heritability

SNP-based heritability estimates from SNPs retained after each AWM analysis were calculated and compared to those from randomly selected SNPs. The variants from each AWM analysis explained 39, 36 and 38% of the phenotypic variance of RFI, RG and FE, respectively, whereas the randomly selected SNPs explained 10, 9 and 14%, respectively.

### Functional analyses

**Fig. 1:**
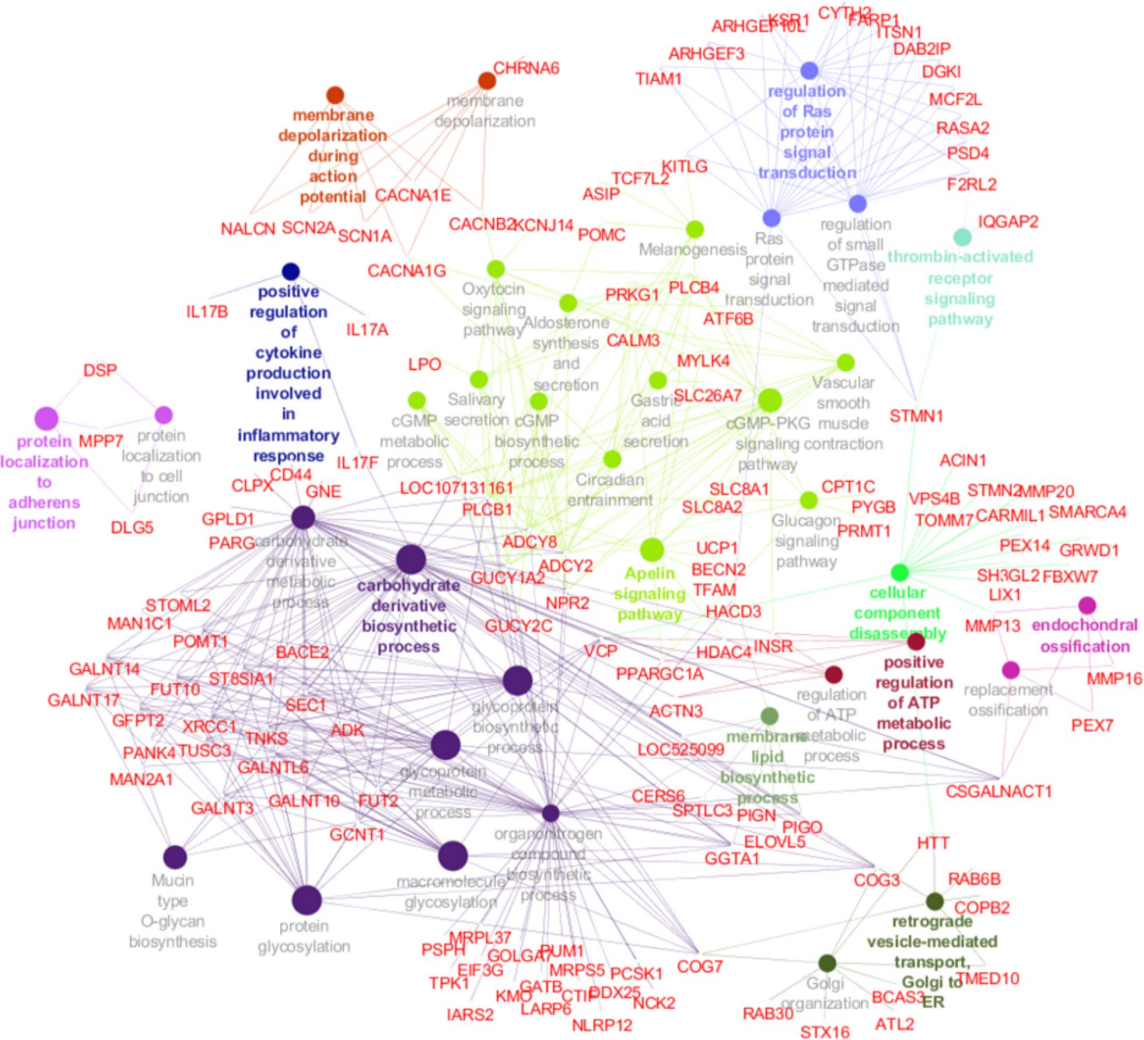
Network interaction between GO terms, KEGG pathways and genes from the association weight matrix (AWM) analysis with RFI as key phenotype

Enrichment results from ClueGO for the three analyses are in Tables S4, S5 and S6 [see Additional file 4: Tables S4, S5 and S6] and the interaction networks between genes and processes are in Figures S2 and S3 [see Additional file 5: Figs. S2 and S3]. The genes retained after the AWM analysis with RFI as key phenotype enriched 11 KEGG pathways and 26 GO terms, which clustered into 12 functional groups ([see Additional file 4: Table S4] and see Fig. 1). On average, the KEGG pathways and GO terms contained 12 genes. The biological process that contained the largest number of genes was “organonitrogen compound biosynthetic process”, which included 55 genes. The “carbohydrate derivative biosynthetic process” was the highest significantly enriched process (P-value = 2.02E−04) (see Table 6). The largest functional group was represented by 10 KEGG pathways and two GO terms. From the AWM analysis with RG as key phenotype, the enrichment analysis highlighted 34 KEGG pathways and 67 GO terms, which were grouped into 27 functional annotations [see Additional file 4: Table S5 and Additional file 5: Fig. S2]. The largest functional group contained 29 KEGG pathways. The biological processes and KEGG pathways included on average six genes and the “regulation of GTPase activity process” was the largest with 19 genes. “Retrograde vesicle-mediated transport, Golgi to ER” was the most significantly enriched process (P-value = 3.00E−04) (see Table 6). For the AWM analysis with FE as key phenotype, 10 KEGG pathways and 36 GO terms that clustered into 17 functional groups, were found [see Additional file 4: Table S6 and Additional file 5: Fig. S3]. In this case, the “organonitrogen compound biosynthetic process” was the largest with 59 genes. One GO term and nine KEGG pathways were included in the largest functional group. On average, each process contained 11 genes and the most enriched process was “glycoprotein biosynthetic” (P-value = 9.39E−06) (see Table 6).

**Table 6:** Top 10 of the GO terms and KEGG pathways enriched by the three feed efficiency networks^a^

GOID	GO term	P-value	% Associated genes	Number of genes
RFI				
GO:1901137	Carbohydrate derivative biosynthetic process	2.02E−04	6.42	35
GO:000900	Glycoprotein metabolic process	2.15E−04	7.74	24
GO:0043413	macromolecule glycosylation	2.61E−04	8.88	19
GO:0006486	Protein glycosylation	2.61E−04	8.88	19
GO:0009101	Glycoprotein biosynthetic process	3.08E−04	8.59	22
GO:1901135	Carbohydrate derivative metabolic process	2.06E−03	4.97	45
GO:0071896	Protein localization to adherens junction	3.47E−03	75.00	3
KEGG:04371	Apelin signaling pathway	3.80E−03	9.15	13
KEGG:00512	Mucin type O-glycan biosynthesis	4.30E−03	19.35	6
KEGG:04022	cGMP-PKG signaling pathway	4.37E−03	8.43	14
RG				
GO:0006890	Retrograde vesicle-mediated transport, Golgi to ER	3.00E−04	20.59	7
GO:0043087	Regulation of GTPase activity	1.99E−03	5.32	19
GO:0043547	Positive regulation of GTPase activity	7.03E−03	5.23	16
GO:0009101	Glycoprotein biosynthetic process	8.65E−03	5.47	14
GO:0008589	Regulation of smoothened signaling pathway	1.26E−02	10.34	6
GO:0009100	Glycoprotein metabolic process	1.28E−02	4.84	15
KEGG:04713	Circadian entrainment	1.32E−02	8.00	8
KEGG:04713	Circadian entrainment	1.32E−02	8.00	8
KEGG:04913	Ovarian steroidogenesis	1.33E−02	10.00	6
GO:0007030	Golgi organization	1.40E−02	8.24	7
FE				
GO:0009101	Glycoprotein biosynthetic process	9.39E−06	8.98	23
GO:0009100	Glycoprotein metabolic process	1.04E−05	8.39	26
GO:1901137	Carbohydrate derivative biosynthetic process	1.31E−05	6.42	35
GO:1901135	Carbohydrate derivative metabolic process	3.36E−05	5.19	47
KEGG:00512	Mucin type O-glycan biosynthesis	3.95E−05	25.81	8
GO:0043413	Macromolecule glycosylation	2.24E−04	8.41	18
GO:0006486	Protein glycosylation	2.24E−04	8.41	18
GO:1901566	Organonitrogen compound biosynthetic process	2.83E−04	4.37	59
GO:0030948	Negative regulation of vascular endothelial growth factor receptor signaling pathway	5.27E−03	60.00	3
KEGG:04971	Gastric acid secretion	1.37E−02	10.81	8

*RFI* residual feed intake, *RG* residual gain, *FE* feed efficiency ratio

**Fig. 2:**
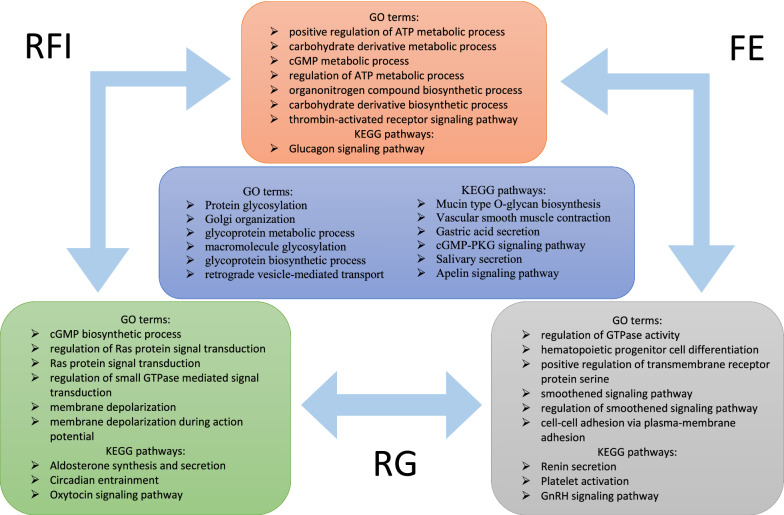
Shared biological processes between the three feed efficiency traits. ^a^Trait abbreviations: *RFI* residual feed intake, *RG* residual gain; *FE* feed efficiency ratio

Six GO terms and six KEGG pathways were shared between the three analyses (see Fig. 2). The six GO terms were related to Golgi (“Golgi organization and retrograde vesicle-mediated transport—Golgi to ER “) and glycosylation (“macromolecule glycosylation, glycoprotein metabolic process, glycoprotein biosynthetic process and protein glycosylation”) processes. Digestive tract processes (“mucin type O-glycan biosynthesis, salivary secretion and gastric acid secretion”), the “cGMP-PKG signaling” and the “vascular smooth muscle contraction and apelin signaling” pathways were associated with the six shared KEGG pathways because of common genes that enriched the same processes. For example, the “gastric acid secretion process” was overrepresented by *ADCY8*, *CALM3*, *PLCB1*, *PLCB4* and *SLC26A7*. RFI and RG shared six GO terms and three KEGG pathways (see Fig. 2), which were related to “membrane depolarization”, “Ras protein”, “small GTPase”, “cGMP biosynthetic process”, “circadian entrainment”, “oxytocin signaling pathway” and “aldosterone”. RFI and FE shared the GO terms “thrombin”, “organonitrogen”, “cGMP”, “ATP” and “carbohydrate” processes and the glucagon signaling KEGG pathway. Positive regulation of the ATP metabolic process was overrepresented by the *ACTN3*, *INSR*, *PPARGC1A* and *VCP* genes. RG and FE shared six GO terms (“related to cell–cell adhesion”, “smoothened signaling pathway”, “transmembrane receptor protein”, “serine/threonine kinase” and “GTPase” activity) and three KEGG pathways (“platelet activation”, “GnRH signaling pathway” and “renin secretion”) (see Fig. 2).

## Discussion

### Identification of several variants with small effects

In the past, most studies have focused their GWAS on a P-value approach, using a Bonferroni or false discovery rate (FDR) threshold. These methods attempt to find the good balance between highly conservative thresholds that result in strong but few SNPs, and relaxed thresholds that result in more SNPs but with potential false positive results. In our study, at the whole-genome scale, this approach would have been too stringent and no SNPs would have passed the thresholds. This absence of major effects for SNPs associated with feed efficiency has already been observed in the literature and, usually, very weak thresholds (in log_10_) are set to detect significant associations. For instance, in [7] 75 SNPs were associated with RFI with a P-value of 0.001; in [8] 31 SNPs were associated with RFI with the same P-value but none exceeded the FDR threshold; in [10] only two SNPs were detected that exceeded the Bonferroni threshold; and in [15] only three quantitative trait loci (QTL) for RFI were reported when using a Bonferroni threshold for high-density SNPs, of which only one reached the whole-genome threshold. These previously published results confirm that feed efficiency is a complex trait that is influenced by several biological processes.

In our study, first we used an approach to select the 100 variants with the lowest p-value for each feed efficiency criterion and to investigate in which gene they were present. Some genes related to proteolysis such as *CAPN7* and *UBQLNL* were highlighted in this approach. *CAPN7* encodes a calpain that is known for its role in meat tenderness in several species. *UBQLNL* is involved in the ubiquitination machinery that regulates the degradation, cellular localization, activation and inactivation of proteins. *CAPN7* and *UBQLNL* are involved in protein turnover, and low RFI animals seem to adopt a low protein degradation strategy to decrease energy expenditure of this function [35]. We also identified *INSL5* that was associated with RG, and *CACNA1E* that was associated with both RFI and FE. *INSL5* is linked to energetic functions and involved in hepatic glucose production [36]. *CACNA1E* encodes the calcium voltage-gated channel subunit alpha1 E, which is one of the subunits that form the channel that mediates the entry of calcium ions into excitable cells. It is involved in a variety of calcium-dependent processes, including muscle contraction, hormone or neurotransmitter release, gene expression, cell motility, cell division and cell death.

### SNPs selected by the AWM approach are informative

The AWM method was proposed as an alternative approach to retain more variants than the P-value approach while minimising the number of false positive variants by in-silico validation [21]. Only a few studies [37, 38] have used this approach for feed efficiency traits. The AWM networks highlighted more genes associated with RFI than with FE and RG. Regardless of the trait, these genes explained a significant part of the phenotypic variance, i.e. 39% for RFI, 36% for RG and 38% for FE. The genomic correlation between RFI and ADG, although quite low (ranging from − 0.29 to − 0.11), was surprising because, by definition, these two traits are phenotypically independent and were genetically independent (− 0.04). In a study on pigs, a very low genomic correlation (0.011) was found between RFI and ADG [38]. Interestingly, the strongest genomic correlations found in this study were between RFI and FE. In the literature, genomic correlations of 0.63 and 0.763 were estimated between RFI and FCR using a bovine 50 K SNP array [37] and in pig [38], respectively.

Five common genes were identified in each of the three feed efficiency networks: *WDR27*, *PCDH8*, *CDCP2*, *ENSBTAG00000030623* and *MROH7*. *ENSBTAG00000030623* and *MROH7* were among the genes that harboured SNPs from the 100 SNPs with the lowest p-value for RG and FE (Table 2). Previous studies reported that *CDCP2*, *ENSBTAG00000030623* and *MROH7* were located within two QTL for body weight and dystocia in Holstein cattle [39] and *PCDH8* in a QTL for marbling score in Angus cattle [40]. We also found that the *NCK2* gene was retained in the two AWM analyses with RFI and FE as key phenotypes and was associated with all three feed efficiency criteria. Moreover, *NCK2* was among the genes that harboured SNPs from the SNPs with the 100 lowest P-value for RG. In Angus cattle, the *NCK2* gene has been located in four QTL for yearling and mature body weight, marbling score and fat thickness [40].

### Biological functions shared with feed efficiency traits

To help interpretation of the underlying biological functions of all the genes identified in the three feed efficiency networks, we performed enrichment analyses of biological processes and metabolic pathways. This work allowed us to confirm the effects of the genes detected by the AWM approach and to better understand the mechanisms involved in each feed efficiency trait. One first result is that the RFI network enriched the smallest number of GO terms and KEGG pathways although it contained the largest number of genes. Conversely, the RG network comprised the smallest number of genes but enriched the largest number of GO terms and KEGG pathways. This suggests that the genes in the RG network might have a higher level of pleiotropy than those in the RFI network, and thus the latter might be more specific. In spite of the differences in the biological processes identified in each network, some were common across the networks (see Fig. 2).

#### Digestive functions

The three feed efficiency networks were enriched for mucin type O-glycan. Mucins are glycoproteins that are secreted in mucosal sites such as the urogenital, airway and gastrointestinal tracts, and are the major macromolecular components of mucus [41]. Mucin type-O-glycan plays roles in cell protection i.e. in mucus barrier functions, and promotes homeostasis with microbes. Dysfunction of the mucin barrier enhances chronic inflammation of the colon in mice devoid of mucin [42]. A study on the human gut showed that mucin O-glycan has an important role in the synthesis of n-butyrate by the microbiota, which regulates T cell differentiation to reduce gut inflammation [43]. The three feed efficiency networks highlighted two KEGG pathways linked to the digestive tract: “salivary secretion” and “gastric acid secretion”. In cattle, saliva does not change the structure of cellulose but enhances cellulase-catalyzed degradation of cellulose [44]. Gastric acid is secreted in the abomasum of cattle and contributes to feed digestion. The “apelin signalling” pathway was also enriched in each network and had pleiotropic functions in hydric and energetic homeostasis, digestive tract, adipo-insular axis, cardiovascular system and angiogenesis. It has been suggested that apelin is related with feed and water intake, digestive mobility, absorption and digestive secretions [45]. A study on goats highlighted the role of apelin in feeding processes and in the pituitary gland for enhancing secretion of the adrenocorticotropic hormone (ACTH) and growth hormone (GH) [46].

#### Energy metabolism

Several processes related to the energetic pathway were enriched by both RFI and FE networks, in particular the regulation of ATP and carbohydrate processes (see Fig. 2). Mitochondria are responsible of approximately 90% of the oxygen consumption [47]. It has been reported that the lymphocytes of low-RFI steers have a larger mitochondrial complex I, which suggests a higher production of ATP in efficient animals [48]. Moreover, the respirator acceptor control ratio (state 3:state 2) was higher in low-RFI beef cattle [49]. In lambs, a negative correlation was found between RFI and respiratory chain complex activities [50]. In addition, high FE broilers had higher complex I and II activities [51]. Taken together, these results confirm our finding that RFI and FE have an energetic function and the important role of mitochondria in feed efficiency, as explained in detail in [52]. Another energetic pathway enriched by both the RFI and FE networks was the glucagon signalling pathway. Glucagon reduces glucose utilisation in adipose tissue, stimulates lipolysis and increases gluconeogenesis. This pathway was also highlighted in Nellore cattle [53] and the *ADCY2* gene was identified in both our study and [53].

#### Vascular system

The “cGMP-PKG signalling” pathway (regulation of relaxation and contraction of vascular smooth muscle cells) and “vascular smooth muscle contraction” pathway were enriched by the three feed efficiency networks. In addition, the RFI and FE networks also included thrombin-activated receptor. Thrombin is a serine protease that regulates platelet aggregation, endothelial cell activation and several processes in vascular biology such as conversion of circulating fibrinogen to fibrin monomer [54]. Linked to this protease, the thrombin signalling pathway was also enriched in a study that performed Ingenuity Pathway Analysis [37]. Moreover, the thrombin receptor signalling pathway was reported in [53] and two of the genes, *F2RL2* and *IQGAP2*, detected in [53], were found in our study. Both the RG and FE networks also enriched several processes related to the vascular system, such as renin secretion, hematopoietic progenitor cell differentiation, smoothened signalling pathway and platelet activation (see Fig. 2). Several other studies also highlighted cardio-vascular system processes associated with feed efficiency [37, 38, 55].

#### Hormones

Both RG and FE networks enriched the gonadotrophin releasing hormone (GnRH) signalling pathway, which is known to be the most significantly enriched KEGG pathway for growth and RFI enriched networks [37]. This pathway has also been shown to be enriched for RFI only [55]. Although no clear relationship has been established between feed efficiency and reproductive functions, a study on lambs immunized against GnRH had lower ADG, higher feed intake and lower FE compared to untreated animals [56]. Another study with Angus cattle revealed a higher GnRH and lower gonadotropin-inhibitory hormone (GnIH) expression level for highly feed efficient animals [57]. These results suggest that GnRH could be involved in feed efficiency and growth regulation. Another interesting result was the enrichment of the oxytocin signalling pathway by both RFI and RG networks. Some studies highlighted oxytocin for its role in the regulation of gastrointestinal motility in rabbits [58] and rats [59]. Moreover, oxytocin was reported to be expressed throughout the human digestive tract [60]. This hormone could have a role in feed efficiency in beef cattle through its function in the digestive process, notably in gastrointestinal motility. Both RFI and RG networks also enriched the aldosterone synthesis and secretion pathway. Aldosterone is a mineralocorticoid hormone involved in sodium–potassium balance and hydric regulation. Aldosterone activity involved in renal sodium reabsorption is associated with feed efficiency in Nellore bulls [61]. A study on piglets estimated that 10% of the metabolizable energy for maintenance is related to mineral reabsorption in the kidney [62]. The aldosterone signalling pathway in epithelial cells was found to be associated with feed efficiency in pigs [38].

#### Other biological functions

Metabolisms related to glycoproteins were enriched in the three feed efficiency networks. Glycoproteins are subjected to post-transcriptional modifications that catalyse covalent cross-linking of oligosaccharide chains to amino acids [41]. Glycoproteins have several functions related to the immune system, transport, reproduction, hormones and protection of cells. Glycoprotein processes take place in the Golgi and glycoproteins are transported in vesicles. Processes related to Golgi organization and retrograde vesicle-mediated transport (Golgi to ER) were also enriched in the three feed efficiency networks. The whole biology of glycoprotein was related to the three feed efficiency criteria (Fig. 2). Ras protein processes were enriched by both RFI and RG networks and have functions in cell proliferation, differentiation and survival. In a review, Ras signalling pathway and Ras-related protein 1 signalling pathway were highlighted as common biological pathways in different RFI studies conducted in beef cattle [35]. We also confirmed their result [35] that the circadian entrainment process was enriched by both RFI and RG networks. A previous study that focused on the circadian evolution of blood plasma cortisol showed that it increased during the night, especially for high feed efficiency animals [63]. Cortisol has important functions notably on fat and protein regulation, carbohydrate metabolism and muscle maintenance.

### Biological functions specific to each feed efficiency trait

Our results show that RFI is specifically associated with the ossification process, inflammatory response, cellular component disassembly, protein localization and melanogenesis. Melanogenesis has been shown to be related to RFI [55, 64]. For RG criteria, the network was enriched for two hormones: insulin and thyroid. Insulin is a hormone responsible for glucose uptake by the liver, skeletal muscle and adipose tissue and is one of the factors that activate the mTORC complex, which has a role in cell growth and proliferation and protein synthesis. An enrichment of insulin secretion with RFI was reported in Angus cattle [55]. The thyroid hormone is involved in both lipolysis and lipogenesis functions, which are related to energetic functions and were also enriched in the RG network. This network also enriched a huge number of processes related to heart function from neuronal action potential to muscle contraction and several cellular processes like mitotic functions, cytoskeleton, regulation of proteolysis, vesicle transport, membrane cell and adherent junctions. Feed efficiency ratio was specifically associated with processes related to the immune and stress response, and to vascular endothelial cell, regulation of skeletal muscle fiber development and MAPK pathways. Several studies [35, 55, 64, 65] have revealed mitogen-activated protein kinase (MAP-K) processes related to feed efficiency traits, which suggest the important role of these processes in these phenotypes.

## Conclusions

Our results revealed both common and specific biological processes associated to RFI, RG and FE and allow a better understanding of the genetic determinism of these feed efficiency criteria. Each of the three gene networks, one for each trait, confirmed the strong association between FE and RFI and between FE and RG and also the weaker association between RFI and RG. The enrichment analysis highlighted the complexity of the genetic architecture of feed efficiency, especially for RG that enriched the largest number of processes. Indeed, a huge number of cardio-vascular and cellular processes were highlighted for this trait and several others such as the immune system, reproduction, protein regulation and signalling systems. Interestingly, RFI and FE seemed to be more associated with energy functions, which confirms the role of the energy metabolism on feed efficiency. The three feed efficiency criteria were also associated with digestive tract processes such as salivary, gastric and mucin secretion. Moreover, our study identified several potential markers that could be used to predict feed efficiency e.g. apelin, glucagon, insulin, aldosterone, GnRH or the thyroid hormone. Our findings confirm the real interest of using gene network analyses to identify genes that have small effects on traits. The effects of these genes need to be tested in genomic evaluation to confirm the potential benefit of using functional SNPs to select animals for feed efficiency.

## Supplementary information

**Additional file 1.** List of breeds included in the reference population panel for whole-genome imputation.

**Additional file 2: Fig. S1.** Manhattan plots of residual feed intake (a), residual gain (b) and feed efficiency ratio (c).

**Additional file 3: Table S1.** List of genes retained after the AWM analysis with RFI as key phenotype. **Table S2.** List of genes retained after the AWM analysis with RG as key phenotype. **Table S3.** List of genes retained after the AWM analysis with FE as key phenotype.

**Additional file 4: Table S4.** List of GO terms and KEGG pathways enriched by genes retained after the AWM analysis with RFI as key phenotype. **Table S5.** List of GO terms and KEGG pathways enriched by genes retained after the AWM analysis with RG as key phenotype. **Table S6.** List of GO terms and KEGG pathways enriched by genes retained after the AWM analysis with FE as key phenotype.

**Additional file 5: Fig. S2.** Network interaction between GO terms, KEGG pathways and genes from the AWM analysis with RG as key phenotype. **Fig. S3.** Network interaction between GO terms, KEGG pathways and genes from the AWM analysis with FE as key phenotype.

## Data Availability

The data (genotypes and phenotypes) that support the findings of this study are available from INRAE but restrictions apply to the availability of these data, which were used under license for the current study, and thus are not publicly available.

## References

[1] Chotteau P, Garrigues B, Cotto G, Guesdon JC, Kempf M (1997). 1996: l’année économique lait et viande bovine perspective 1997.

[2] Dimon P, Blachon A, Lapostolle L, Lomelet B, Oden D, Lecomte C. Résultats 2016 des élevages BV suivis par Bovins Croissance. 2017. Paris: Institut de l'élevage; 2017. https://idele.fr/contact/publication/idelesolr/recommends/resultats-2016-des-elevages-bovins-viande-suivis-par-bovins-croissance.html. Accessed 8 August 2018.

[3] Buczinski B, Bechet E, Benoteau G, Galisson B, Carteron P, Guibert R.. Vaches, surfaces, charges… tout augmente sauf le revenu. Paris: Institut de l'élevage; 2016. https://idele.fr/no_cache/recherche/publication/idelesolr/recommends/vaches-surfaces-charges-tout-augmente-sauf-le-revenu.html. Accessed 9 August 2018.

[4] Zetouni L, Henryon M, Kargo M, Lassen J (2017). Direct multitrait selection realizes the highest genetic response for ratio traits. J Anim Sci.

[5] Koch RM, Swiger LA, Chambers D, Gregory KE (1963). Efficiency of feed use in beef cattle. J Anim Sci.

[6] Berry DP, Crowley JJ (2013). Cell biology symposium: genetics of feed efficiency in dairy and beef cattle. J Anim Sci.

[7] Bolormaa S, Hayes BJ, Savin K, Hawken R, Barendse W, Arthur PF (2011). Genome-wide association studies for feedlot and growth traits in cattle. J Anim Sci.

[8] Lu D, Miller S, Sargolzaei M, Kelly M, Vander Voort G, Caldwell T (2013). Genome-wide association analyses for growth and feed efficiency traits in beef cattle. J Anim Sci.

[9] Saatchi M, Beever JE, Decker JE, Faulkner DB, Freetly HC, Hansen SL (2014). QTLs associated with dry matter intake, metabolic mid-test weight, growth and feed efficiency have little overlap across 4 beef cattle studies. BMC Genomics.

[10] Santana MHA, Utsunomiya YT, Neves HHR, Gomes RC, Garcia JF, Fukumasu H (2014). Genome-wide association analysis of feed intake and residual feed intake in Nellore cattle. BMC Genet.

[11] de Oliveira PSN, Cesar ASM, do Nascimento ML, Chaves AS, Tizioto PC, Tullio RR (2014). Identification of genomic regions associated with feed efficiency in Nelore cattle. BMC Genet.

[12] Olivieri BF, Mercadante MEZ, Cyrillo JNDSG, Branco RH, Bonilha SFM, de Albuquerque LG (2016). Genomic regions associated with feed efficiency indicator traits in an experimental Nellore cattle population. PLoS One.

[13] Seabury CM, Oldeschulte DL, Saatchi M, Beever JE, Decker JE, Halley YA (2017). Genome-wide association study for feed efficiency and growth traits in U.S. beef cattle. BMC Genomics.

[14] Higgins MG, Fitzsimons C, McClure MC, McKenna C, Conroy S, Kenny DA (2018). GWAS and eQTL analysis identifies a SNP associated with both residual feed intake and GFRA2 expression in beef cattle. Sci Rep.

[15] Martin P, Taussat S, Vinet A, Krauss D, Maupetit D, Renand G (2019). Genetic parameters and genome-wide association study regarding feed efficiency and slaughter traits in Charolais cows. J Anim Sci.

[16] Lu D, Akanno EC, Crowley JJ, Schenkel F, Li H, De Pauw M (2016). Accuracy of genomic predictions for feed efficiency traits of beef cattle using 50K and imputed HD genotypes. J Anim Sci.

[17] Meuwissen T, Goddard M (2010). Accurate prediction of genetic values for complex traits by whole-genome resequencing. Genetics.

[18] Daetwyler HD, Capitan A, Pausch H, Stothard P, van Binsbergen R, Brøndum RF (2014). Whole-genome sequencing of 234 bulls facilitates mapping of monogenic and complex traits in cattle. Nat Genet.

[19] Fitzsimons C, McGee M, Keogh K, Waters SM, Kenny DA, Scanes CG, Hill RA (2017). Molecular physiology of feed efficiency in beef cattle. Biology of domestic animals.

[20] Teissier M, Sanchez MP, Boussaha M, Barbat A, Hoze C, Robert-Granie C (2018). Use of meta-analyses and joint analyses to select variants in whole genome sequences for genomic evaluation: An application in milk production of French dairy cattle breeds. J Dairy Sci.

[21] Fortes MRS, Reverter A, Zhang Y, Collis E, Nagaraj SH, Jonsson NN (2010). Association weight matrix for the genetic dissection of puberty in beef cattle. Proc Natl Acad Sci USA.

[22] Snelling WM, Cushman RA, Keele JW, Maltecca C, Thomas MG, Fortes MRS (2013). Breeding and Genetics Symposium: Networks and pathways to guide genomic selection. J Anim Sci.

[23] Ramayo-Caldas Y, Renand G, Ballester M, Saintilan R, Rocha D (2016). Multi-breed and multi-trait co-association analysis of meat tenderness and other meat quality traits in three French beef cattle breeds. Genet Sel Evol.

[24] Pegolo S, Mach N, Ramayo-Caldas Y, Schiavon S, Bittante G, Cecchinato A (2018). Integration of GWAS, pathway and network analyses reveals novel mechanistic insights into the synthesis of milk proteins in dairy cows. Sci Rep.

[25] Taussat S, Saintilan R, Krauss D, Maupetit D, Fouilloux MN, Renand G (2019). Relationship between feed efficiency and slaughter traits of French Charolais bulls. J Anim Sci.

[26] Boichard D, Guillaume F, Baur A, Croiseau P, Rossignol MN, Boscher MY (2012). Genomic selection in French dairy cattle. Anim Prod Sci.

[27] Sanchez MP, Ramayo-Caldas Y, Wolf V, Laithier C, El Jabri M, Michenet A (2019). Sequence-based GWAS, network and pathway analyses reveal genes co-associated with milk cheese-making properties and milk composition in Montbéliarde cows. Genet Sel Evol.

[28] van Binsbergen R, Bink MC, Calus MP, van Eeuwijk FA, Hayes BJ, Hulsegge I (2014). Accuracy of imputation to whole-genome sequence data in Holstein Friesian cattle. Genet Sel Evol.

[29] Sargolzaei M, Chesnais JP, Schenkel FS (2014). A new approach for efficient genotype imputation using information from relatives. BMC Genomics.

[30] Howie B, Fuchsberger C, Stephens M, Marchini J, Abecasis GR (2012). Fast and accurate genotype imputation in genome-wide association studies through pre-phasing. Nat Genet.

[31] Yang J, Lee SH, Goddard ME, Visscher PM (2011). GCTA: a tool for genome-wide complex trait analysis. Am J Hum Genet.

[32] Yang J, Benyamin B, McEvoy BP, Gordon S, Henders AK, Nyholt DR (2010). Common SNPs explain a large proportion of the heritability for human height. Nat Genet.

[33] Bindea G, Mlecnik B, Hackl H, Charoentong P, Tosolini M, Kirilovsky A (2009). ClueGO: a Cytoscape plug-in to decipher functionally grouped gene ontology and pathway annotation networks. Bioinformatics.

[34] Bindea G, Galon J, Mlecnik B (2013). CluePedia Cytoscape plugin: pathway insights using integrated experimental and in silico data. Bioinformatics.

[35] Cantalapiedra-Hijar G, Abo-Ismail M, Carstens GE, Guan LL, Hegarty R, Kenny DA (2018). Review: biological determinants of between-animal variation in feed efficiency of growing beef cattle. Animal.

[36] Lee YS, De Vadder F, Tremaroli V, Wichmann A, Mithieux G, Bäckhed F (2016). Insulin-like peptide 5 is a microbially regulated peptide that promotes hepatic glucose production. Mol Metab.

[37] Widmann P, Reverter A, Weikard R, Suhre K, Hammon HM, Albrecht E (2015). Systems biology analysis merging phenotype, metabolomic and genomic data identifies non-SMC condensin I complex, subunit G (NCAPG) and cellular maintenance processes as major cntributors to genetic variability in bovine feed efficiency. PLoS One.

[38] Ramayo-Caldas Y, Mármol-Sánchez E, Ballester M, Sánchez JP, González-Prendes R, Amills M (2019). Integrating genome-wide co-association and gene expression to identify putative regulators and predictors of feed efficiency in pigs. Genet Sel Evol.

[39] Sugimoto M, Watanabe T, Sugimoto Y (2012). The molecular effects of a polymorphism in the 5′UTR of *solute carrier family 44, member 5* that is associated with birth weight in Holsteins. PLoS One.

[40] McClure MC, Morsci NS, Schnabel RD, Kim JW, Yao P, Rolf MM (2010). A genome scan for quantitative trait loci influencing carcass, post-natal growth and reproductive traits in commercial Angus cattle. Anim Genet.

[41] Bergstrom KSB, Xia L (2013). Mucin-type O-glycans and their roles in intestinal homeostasis. Glycobiology.

[42] Sommer F, Adam N, Johansson MEV, Xia L, Hansson GC, Bäckhed F (2014). Altered mucus glycosylation in core 1 O-glycan-deficient mice affects microbiota composition and intestinal architecture. PLoS One.

[43] Yamada T, Hino S, Iijima H, Genda T, Aoki R, Nagata R (2019). Mucin O-glycans facilitate symbiosynthesis to maintain gut immune homeostasis. EBioMedicine.

[44] Seki Y, Kikuchi Y, Kimura Y, Yoshimoto R, Takahashi M, Aburai K (2015). Enhancement of cellulose degradation by cattle saliva. PLoS One.

[45] Picault FX. Signalisation apeline et adénocarcinomes coliques. PhD thesis. Université de Toulouse; 2013. https://thesesups.ups-tlse.fr/2407/. Accessed 3 December 2019.

[46] Sato K, Takahashi T, Kobayashi Y, Hagino A, Roh SG, Katoh K (2012). Apelin is involved in postprandial responses and stimulates secretion of arginine-vasopressin, adrenocorticotropic hormone, and growth hormone in the ruminant. Domest Anim Endocrinol.

[47] Bottje WG, Carstens GE, Hill RA (2012). Variation in metabolism: biological efficiency of energy production and utilization that affects feed efficiency. Feed efficiency in the beef industry.

[48] Ramos MH, Kerley MS (2013). Mitochondrial complex I protein differs among residual feed intake phenotype in beef cattle. J Anim Sci.

[49] Lancaster PA, Carstens GE, Michal JJ, Brennan KM, Johnson KA, Davis ME (2014). Relationships between residual feed intake and hepatic mitochondrial function in growing beef cattle. J Anim Sci.

[50] Sharifabadi HR, Zamiri MJ, Rowghani E, Bottje WG (2012). Relationship between the activity of mitochondrial respiratory chain complexes and feed efficiency in fat-tailed Ghezel lambs. J Anim Sci.

[51] Bottje W, Iqbal M, Tang ZX, Cawthon D, Okimoto R, Wing T (2002). Association of mitochondrial function with feed efficiency within a single genetic line of male broilers. Poult Sci.

[52] Hudson NJ, Bottje WG, Hawken RJ, Kong B, Okimoto R, Reverter A (2017). Mitochondrial metabolism: a driver of energy utilisation and product quality?. Anim Prod Sci.

[53] Brunes LC, Baldi F, Lopes FB, Lôbo RB, Espigolan R, Costa MFO (2020). Weighted single-step genome-wide association study and pathway analyses for feed efficiency traits in Nellore cattle. J Anim Breed Genet.

[54] Coughlin SR (2000). Thrombin signalling and protease-activated receptors. Nature.

[55] Rolf MM, Taylor JF, Schnabel RD, McKay SD, McClure MC, Northcutt SL (2012). Genome-wide association analysis for feed efficiency in Angus cattle. Anim Genet.

[56] Kiyma Z, Adams TE, Hess BW, Riley ML, Murdoch WJ, Moss GE (2000). Gonadal function, sexual behavior, feedlot performance, and carcass traits of ram lambs actively immunized against GnRH. J Anim Sci.

[57] Perkins SD, Key CN, Garrett CF, Foradori CD, Bratcher CL, Kriese-Anderson LA (2014). Residual feed intake studies in Angus-sired cattle reveal a potential role for hypothalamic gene expression in regulating feed efficiency. J Anim Sci.

[58] Li L, Kong X, Liu H, Liu C (2007). Systemic oxytocin and vasopressin excite gastrointestinal motility through oxytocin receptor in rabbits. Neurogastroenterol Motil.

[59] Qin J, Feng M, Wang C, Ye Y, Wang PS, Liu C (2009). Oxytocin receptor expressed on the smooth muscle mediates the excitatory effect of oxytocin on gastric motility in rats. Neurogastroenterol Motil.

[60] Monstein H-J, Grahn N, Truedsson M, Ohlsson B (2004). Oxytocin and oxytocin-receptor mRNA expression in the human gastrointestinal tract: a polymerase chain reaction study. Regul Pept.

[61] Santana MHA, Freua MC, Do DN, Ventura RV, Kadarmideen HN, Ferraz JBS (2016). Systems genetics and genome-wide association approaches for analysis of feed intake, feed efficiency, and performance in beef cattle. Genet Mol Res.

[62] Kies AK, Gerrits WJJ, Schrama JW, Heetkamp MJW, van der Linden KL, Zandstra T (2005). Mineral absorption and excretion as affected by microbial phytase, and their effect on energy metabolism in young piglets. J Nutr.

[63] Montanholi YR, Palme R, Haas LS, Swanson KC, Vander Voort G, Miller SP (2013). On the relationships between glucocorticoids and feed efficiency in beef cattle. Livest Sci.

[64] Abo-Ismail MK, Vander Voort G, Squires JJ, Swanson KC, Mandell IB, Liao X (2014). Single nucleotide polymorphisms for feed efficiency and performance in crossbred beef cattle. BMC Genet.

[65] Serão NV, González-Peña D, Beever JE, Faulkner DB, Southey BR, Rodriguez-Zas SL (2013). Single nucleotide polymorphisms and haplotypes associated with feed efficiency in beef cattle. BMC Genet.

