# Supplementary material for: Gene networks for three feed efficiency criteria reveal shared and specific biological processes
Source: Genet Sel Evol. 2020 Nov 10;52:67. doi: 10.1186/s12711-020-00585-z (PMC7653997; doi:10.1186/s12711-020-00585-z)
Supplement: Supplementary file 1 — Additional file 1. List of breeds included in the reference population panel for whole-genome imputation. [file 12711_2020_585_MOESM1_ESM.docx]

**List of breeds included in reference population panel for whole-genome imputation:**

Abondance

Aubrac

Blonde D'Aquitaine

Brown Swiss

Charolaise

Hereford

Holstein

Limousine

Meuse-Rhine-Yssel

Montbeliarde

Normande

Parthenaise

Red Holstein

Salers

Simmental

Tarentaise

UMC

Vosgienne
