# Supplementary material for: Gene networks for three feed efficiency criteria reveal shared and specific biological processes
Source: Genet Sel Evol. 2020 Nov 10;52:67. doi: 10.1186/s12711-020-00585-z (PMC7653997; doi:10.1186/s12711-020-00585-z)
Supplement: Supplementary file 2 — Additional file 2: Fig. S1. Manhattan plots of residual feed intake (a), residual gain (b) and feed efficiency ratio (c). [file 12711_2020_585_MOESM2_ESM.docx]

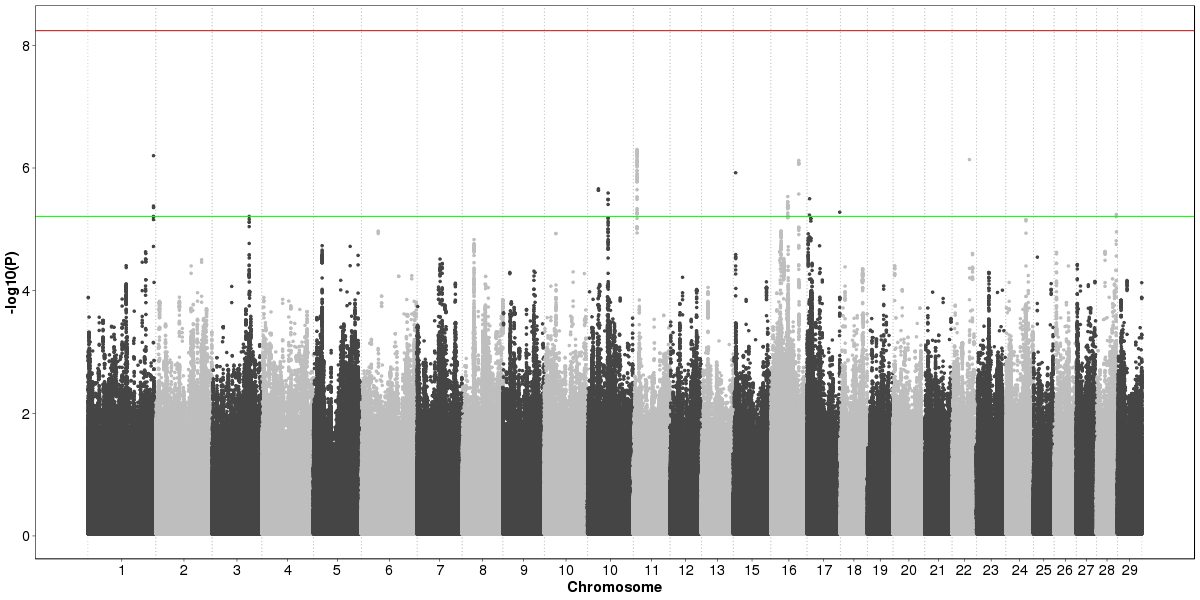


a


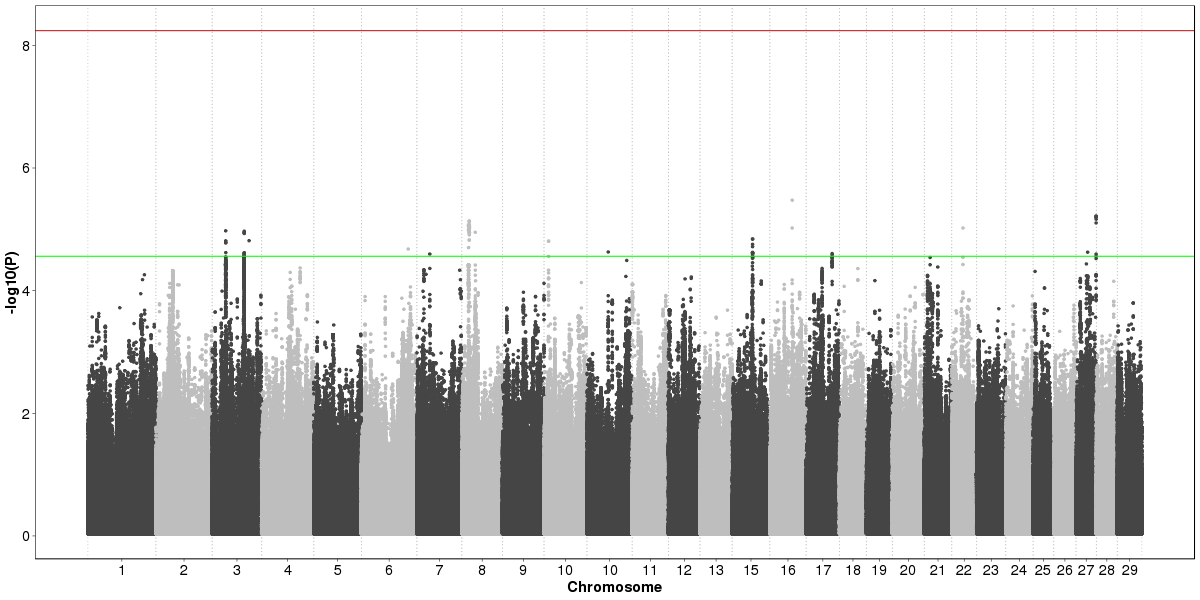


b


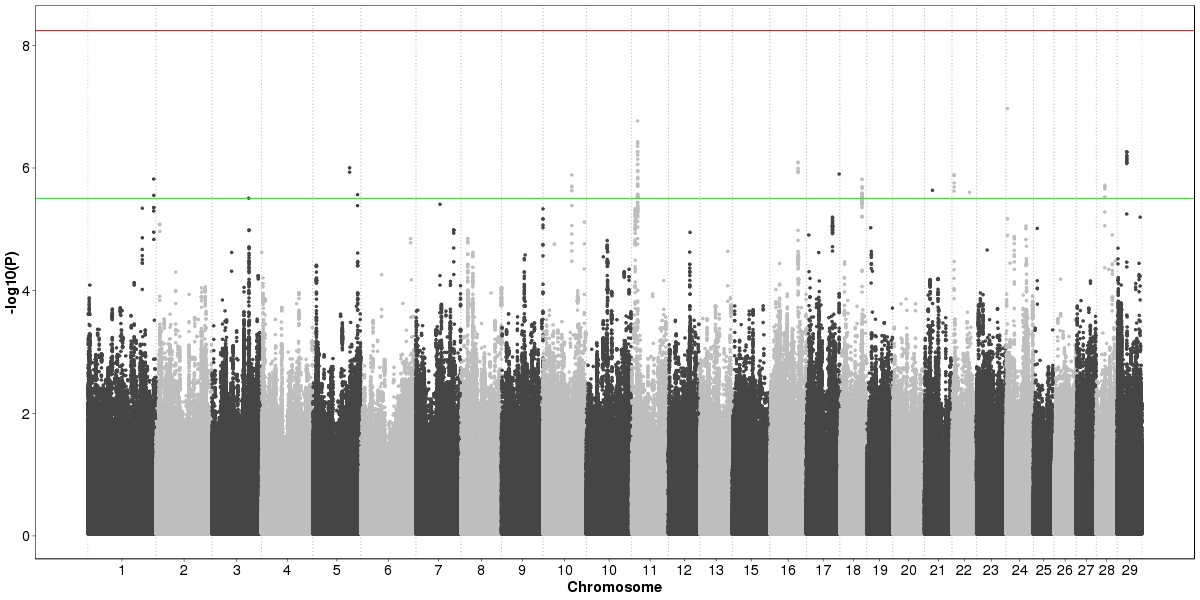


c

**Figure S1 Manhattan plots of residual feed intake (a), residual gain (b) and feed efficiency ratio (c)**

Red line denotes the Bonferroni correction threshold for genome-wide type I error rate of 5% and green line represents the top 100 SNP with the highest –log10(P).
