# Supplementary material for: Gene networks for three feed efficiency criteria reveal shared and specific biological processes
Source: Genet Sel Evol. 2020 Nov 10;52:67. doi: 10.1186/s12711-020-00585-z (PMC7653997; doi:10.1186/s12711-020-00585-z)
Supplement: Supplementary file 5 — Additional file 5: Fig. S2. Network interaction between GO terms, KEGG pathways and genes from the AWM analysis with RG as key phenotype. Fig. S3. Network interaction between GO terms, KEGG pathways and genes from the AWM analysis with FE as key phenotype. [file 12711_2020_585_MOESM5_ESM.docx]

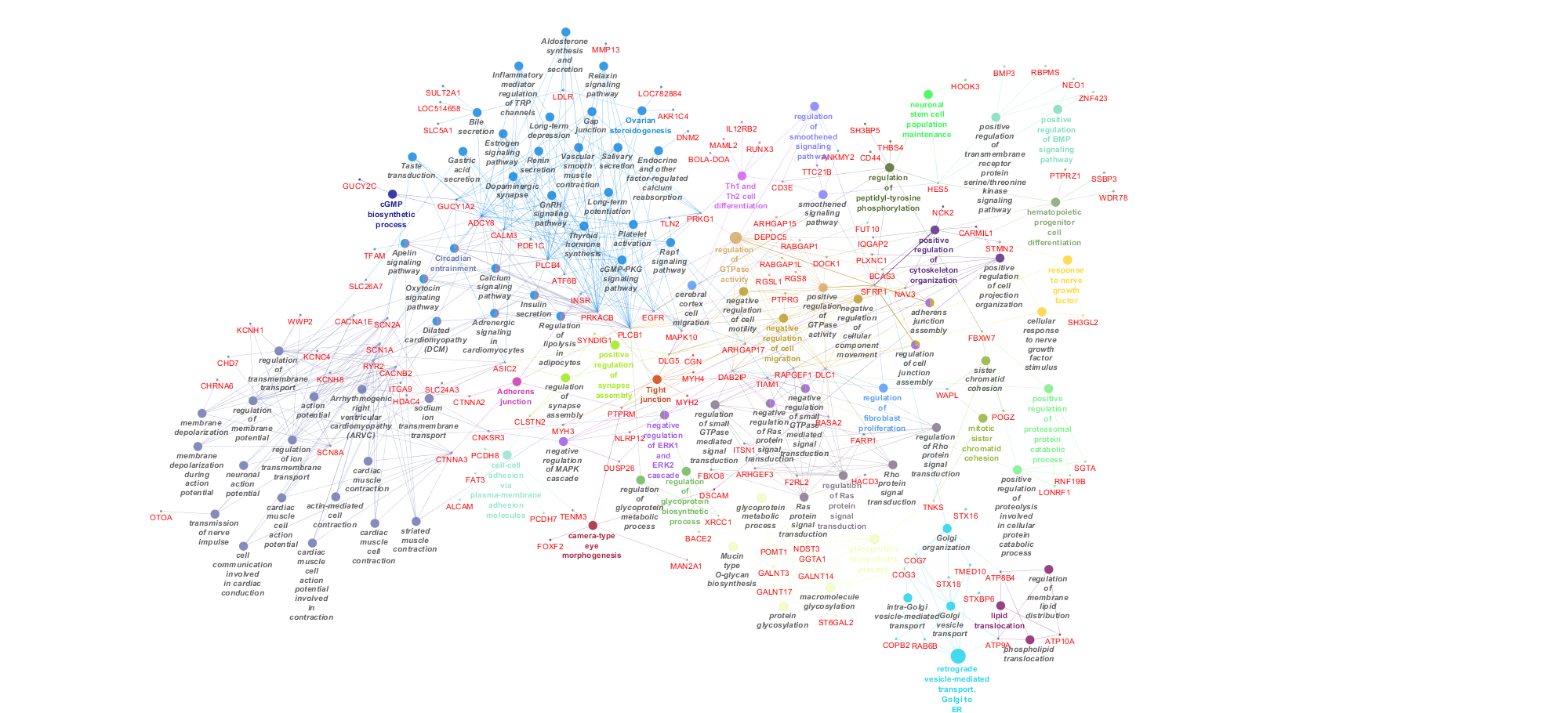


**Figure S2 Network interaction between GO terms, KEGG pathways and genes from the AWM analysis with RG as key phenotype**


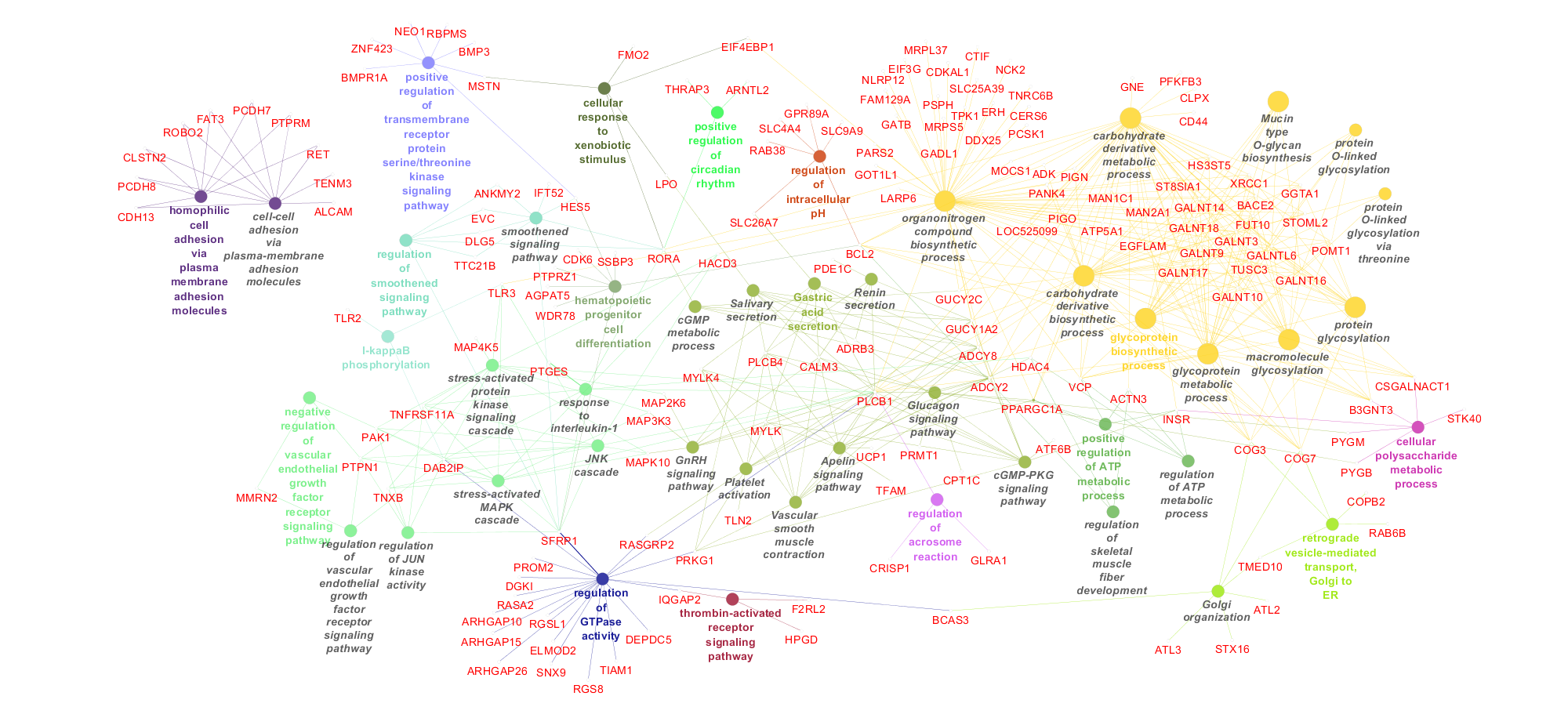


**Figure S3 Network interaction between GO terms, KEGG pathways and genes from the AWM analysis with FE as key phenotype**
